# Flies and Dysentery

**Published:** 1919-11-15

**Authors:** 


### FLIES AND DYSENTERY.

THE report recently presented to the Medical Research Committee by Colonel Leonard Dudgeon provides some yet stronger strictly scientific evidence for connecting fly prevalence with the presence and spread of dysentery.

### IN MACEDONIA.

The subject dealt with is, in this instance, bacillary dysentery as encountered in Macedonia, and we may take the main lines of experiment as they occur. First, the number of flies present at the various seasons in a certain area is compared with relative prevalence of dysentery in that area, and the curves representing the observations made definitely prove that both during April, May, and June, when the fly nuisance is at its height, and during September and October, when the fly prevalence again rises sharply, the number of cases of bacillary dysentery showed an almost exactly corresponding increase. This fact established, attention is turned to experiments proving that a fly, when deliberately infected with bacilli of the dysentery group, is able to carry the organism to a distance and thus transplant the infection elsewhere.

### TYPES OF GERM CARRIED.

In this connection it is now established that the insects can become the vehicle of both the Flexner and Shiga types of germ, and to those not already versed in these methods of investigation some details of the procedure will be of interest. Flies trapped under normal conditions and showing no signs of dysentery germs were kept in specially constructed cages, into which food consisting of milk cultures of the bacilli or other known infected material was passed on a watch glass. After feeding, the flies were examined by various means, such as allowing them to walk on culture plates, killing them and making cultures from various parts of the body, and also examining the excreta.

In a high percentage, in fact nearly 50 per cent. of the insects examined in less than twenty-four hours from their last contact with the infection, a positive result was obtained; but as the time elapsed became greater the positive percentage rapidly decreased. It is establishd, therefore, that flies can readily carry both types of dysentery, but an interesting point arises with the fact that the chances of recovering the organism from their bodies rapidly diminishes at and after twenty-four hours from the time of infection. The true bearing of this latter observation we must leave, for it needs still further elucidation; but the final set of experiments are conclusive and of great importance. For these, insects were caught at random in the hospital wards, kitchen, and latrines at times when the possibility of their previous infection definitely existed. In a great number positive results were obtained, and thus it was proved beyond doubt that flies under natural conditions can carry the bacilli and are capable of transmitting the infection.

### THE LESSON DERIVED.

In previous notes we have called attention to the proved distances which can be traversed by the common flies, and also to sundry methods for the insects' direct or indirect destruction. There is no need to re-traverse that ground, but, if support be yet needed thoroughly to urge the importance of anti-fly campaigns, we have it in ample measure from the findings of these observers. Not with dysentery bacilli alone does the importance lie, but from many other members of the group of coliform bacilli, whose potential power for evil is well known and all too frequently manifest in every country.

